# Early onset rapidly progressive frontotemporal dementia due to a novel MAPT P301A variant with functional validation of pathogenicity

**DOI:** 10.1038/s44400-026-00068-w

**Published:** 2026-05-25

**Authors:** Kiet Hua, Maryam R. Pearson, Brandon I. Apresa, Jesseca M. Chung, Andrew J. Ambrose, Susan M. Hiatt, Argentina Lario Lago, Matthew GH Hall, Liya Rabkina, Stephanie Kwan, Joyce Do, Iyas Daghlas, Annie McDonough, Elan L. Guterman, Denise J. Xu, Howard J. Rosen, Jane Grimwood, J. Nicholas Cochran, Michelle R. Arkin, Adam L. Boxer, Jennifer S. Yokoyama, Peter A. Ljubenkov, Brandon B. Holmes

**Affiliations:** 1 Edward and Pearl Fein Memory and Aging Center, University of California, San Francisco, San Francisco, CA USA; 2 Department of Pharmaceutical Chemistry, University of California, San Francisco, San Francisco, CA USA; 3 Department of Neurology, University of California, San Francisco, San Francisco, CA USA; 4 HudsonAlpha Institute for Biotechnology, Huntsville, AL USA; 5 Department of Neurology, University of Pennsylvania, Philadelphia, PA USA; 6 Weill Institute for Neurosciences, University of California, San Francisco, San Francisco, CA USA; 7 Department of Radiology and Biomedical Imaging, University of California, San Francisco, San Francisco, CA USA

**Keywords:** Diseases, Genetics, Neurology, Neuroscience

## Abstract

Frontotemporal dementia represents a group of clinical syndromes characterized by progressive changes in behavior or language that are associated with frontotemporal lobe atrophy. Around 40% of FTD cases are familial, with mutations in *MAPT* representing a major familial form. Among *MAPT* variants, substitutions at codon 301 – notably P301L, P301S, and P301T – are established pathogenic causes of autosomal-dominant frontotemporal lobar degeneration (FTLD). In contrast, a P301A substitution has not, to our knowledge, been reported in patients. Here, we describe a novel P301A *MAPT* variant presenting with early onset, rapidly progressive FTLD. We show, using biochemical and cellular assays, that P301A tau fibrillizes faster and seeds more robustly than wild-type and P301L tau, with performance comparable to P301S. These findings suggest the P301A variant is pathogenic. This work expands the *MAPT* variant spectrum in FTLD, and supports a model in which distinct substitutions at P301 differentially influence tau aggregation and prion-like propagation.

## Introduction

Frontotemporal dementia (FTD) is a heterogeneous group of syndromes with progressive behavioral or language changes typically associated with frontal or temporal lobe atrophy^1^. FTD is a common cause of young-onset dementia^2,3^. It is a highly heritable condition in which 40% have some family history, with pathogenic variants in *MAPT*, *GRN*, and *C9orf72*, accounting for most of the monogenic cases^4^. In particular, *MAPT* variants directly implicate tau dysfunction in disease pathogenesis. Pathogenic *MAPT* variants include missense, silent, or deletion mutations and occur within exons or introns^5^. Missense substitutions at codon 301 (P301), especially P301L and P301S, are clearly pathogenic causes of autosomal-dominant FTD and are widely used in preclinical models^6–10^.

Here we report a previously undescribed *MAPT* substitution, P301A, in a patient with early onset, rapidly progressive FTD and present complementary biochemical and cell-based studies comparing wild-type tau with P301A, P301L, and P301S. We find that P301A accelerates tau aggregation and increases seeding activity relative to wild-type and established P301 variants. These data expand the genetic spectrum of FTLD-MAPT and highlight P301 as a biophysically sensitive locus where distinct substitutions differentially tune tau pathogenicity.

## Results

### Clinical case description

**Fig. 1:**
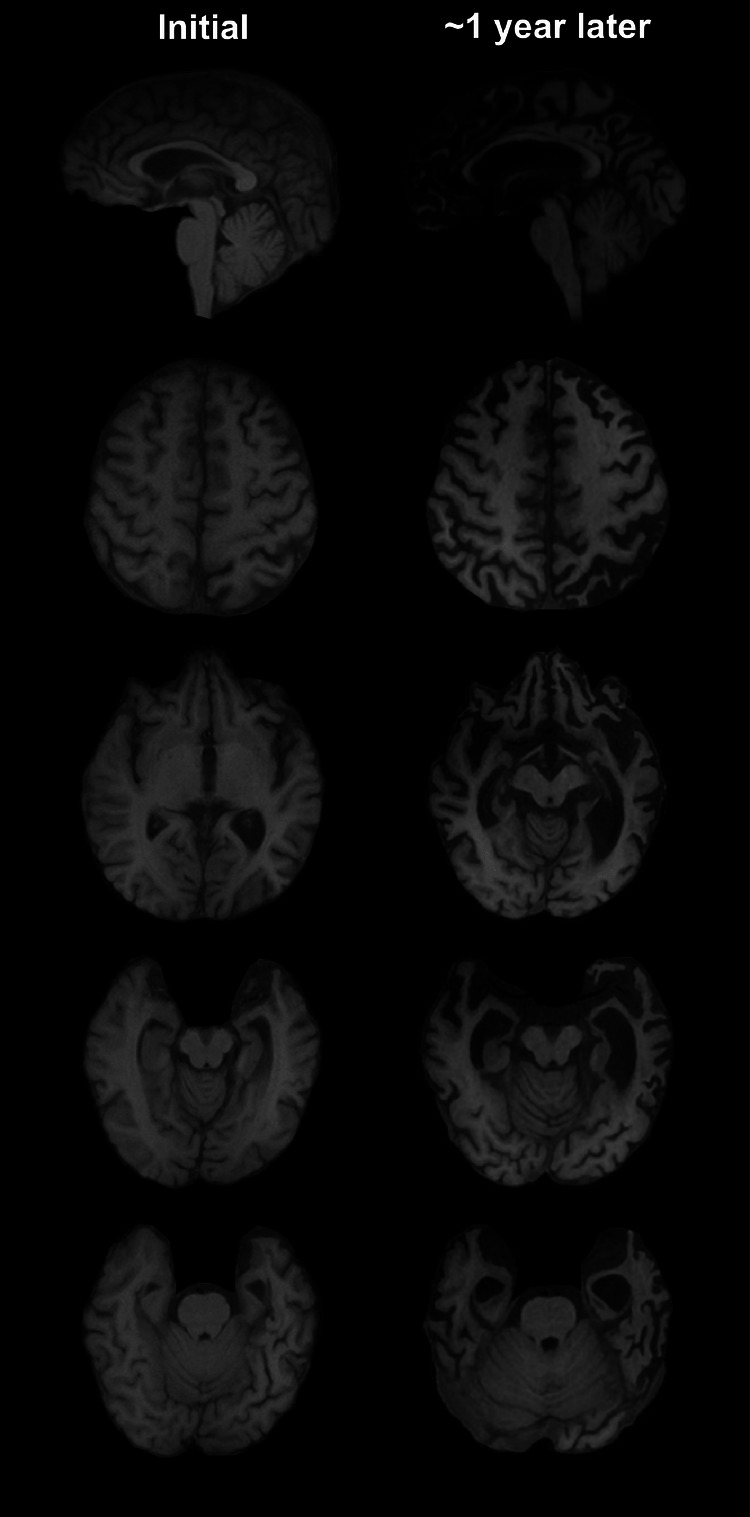
MRI reveals rapidly progressive brain atrophy. Outside brain imaging was compared to follow-up brain MRI (via a 3-T scanner). The initial MRI shows subtle left greater than right amygdala and mesial temporal atrophy. An MRI done approximately 1 year later shows progressive knife-edge atrophy now involving bilateral hemispheres, with left greater than right involvement.

At baseline, the proband is a neurodevelopmentally typical right-hand dominant woman of Southern Chinese descent with a history of strong academic and athletic performance until symptom onset while in college. She first presented to the student health clinic at age 21 with complaints of headache, mild memory symptoms, and difficulty comprehending written text days after being hit in the head with a basketball. There was no loss of consciousness, and she was initially diagnosed with a concussion. A brain MRI performed 3 months after symptom onset, on retrospective review, revealed mild asymmetric temporal lobe volume loss with relatively low amygdala and anterior hippocampus volume (Fig. 1). By 4 months after onset, she lost functional independence, had difficulty navigating familiar areas, and needed assistance with managing appointments. She developed difficulty understanding written and spoken language, anxiety, insomnia, impaired concentration, and irritability.

She was first evaluated by an outside hospital neurologist approximately 6 months after symptom onset. At that visit, she had clear and fluent speech, but exhibited marked distractibility that limited the examination. Review of her outpatient workup notes was significant for perseveration, repeated muttering of profanities, difficulty following conversation, inability to answer questions correctly, and episodes of speaking to herself. She was given various diagnoses, including prolonged concussion syndrome, traumatic brain injury, and severe stress reaction.

Eleven months after symptom onset, she developed worsening psychosis, auditory hallucinations, severe apathy, limited social reciprocity, profound loss of receptive language, and her language became restricted to echolalia or perseverative phrases. She also lost independence in activities of daily living and became incontinent.

Fourteen months after symptom onset, she was admitted to the University of California, San Francisco, for further evaluation following continued functional decline. A brain MRI demonstrated progressive severe left greater than right atrophy, most notable in the amygdalae, hippocampi, insular cortices, anterior temporal lobes, dorsal superior frontal lobes, and dorsal parietal lobes (Fig. 1). The MRI did not show evidence of cortical ribboning or diffusion restriction on B1000. There were T2 FLAIR hyperintensities periventricularly and in the bilateral subcortical white matter that was notable in the left greater than right frontal and parietal regions (Fig. S1). Video EEG monitoring showed occasional mild voltage asymmetry with higher voltages on the right hemisphere, which was thought to reflect her asymmetric cortical atrophy.

### Laboratory and genetic testing

Initial cerebrospinal fluid (CSF) studies were acellular with normal levels of protein, LDH, measles antibody, VZV DNA, and pyruvate; gram/bacterial stains were negative. CSF autoimmune encephalopathy (Mayo ENC2) and serum autoimmune/paraneoplastic encephalopathy panels (Mayo ENS2) were negative. Taken together, the absence of diffusion restriction or cortical ribboning on DWI, along with the clinical course and EEG findings, made a diagnosis of prion disease unlikely. Her family then declined additional lumbar punctures, limiting further CSF analysis. As a result, CSF biomarkers for prion disease (including RT-QuIC and 14-3-3 protein), Alzheimer’s disease (Aβ, total tau, phosphorylated tau), and oligoclonal bands were not obtained.

Serum studies were negative for measles antibody, MaTa antibody, neutrophil cytoplasmic antibodies, double-stranded DNA antibody, anti-nuclear antibodies, Sjogren’s antibodies, thyroglobulin antibodies, and thyroperoxidase antibodies. Normal serum levels were detected for ammonia, IgG index, ceruloplasmin, copper, ESR, and CRP. Metabolic (Stanford acylcarnitine profile, Stanford Urine Organic Acids, Stanford Plasma Amino Acids, Mayo AGU20), heavy metal testing, and lysosomal disorder testing (per blood ultrastructural analysis) were also negative.

**Fig. 2:**
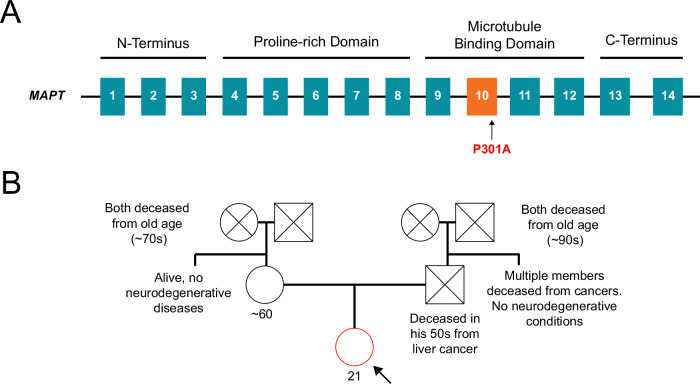
Novel P301A *MAPT* mutation and genetic pedigree. **A** Whole exome sequencing and long-read sequencing in our proband revealed a novel P301A mutation in exon 10 of microtubule-associated protein tau (*MAPT*). **B** A genetic evaluation did not identify any cases of dementia or neurodegenerative disease in the maternal or paternal lineages.

Genetic testing done at UCSF’s Genomic Medicine Lab, including exome sequencing, copy number variant analysis, and mitochondrial genome sequencing, revealed a likely pathogenic heterozygous *MAPT* missense variant: NC_000017.11:g.46010388C>G; NM_005910.6:c.901C>G; NP_005901.2:p.Pro301Ala (Fig. 2A). Focused C9orf72 genetic testing was negative for repeat expansions (repeats = 2, 7). Research long-read sequencing (PacBio HiFi) confirmed the presence of the MAPT c.901C>G (p.Pro301Ala) variant, did not identify any additional single-nucleotide variants, repeat expansions, structural variants, or variants of uncertain significance relevant to her phenotype, and additionally permitted assignment of the MAPT haplotype, which was determined to be H1m. The family history was negative for young-onset dementia, and neither parent was reported to have cognitive impairment (the proband’s father passed away from cancer around age fifty, and the mother was around age sixty at this time) (Fig. 2B). A clinical diagnosis of FTD was made based on advanced behavioral and language decline, meeting diagnostic features of behavioral variant FTD, and primary progressive aphasia^11,12^. The diagnosis was further supported by her *MAPT* variant and MRI pattern of frontal and temporal lobe atrophy. She was subsequently discharged to memory care. By 15 months after symptom onset, she became bedbound due to apathy and, within 2 years, progressed to akinetic mutism with severe bruxism.

### Biochemical and cellular assays

**Fig. 3:**
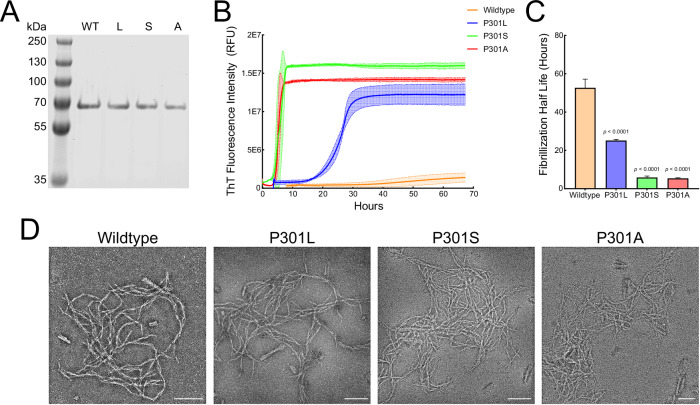
Biochemical characterization of the novel P301A tau mutation. **A** SDS-PAGE analysis of purified full-length 2N4R tau proteins (WT, P301L, P301S, and P301A) demonstrates high purity of each preparation. **B** Thioflavin T (ThT) kinetic aggregation assays reveal that P301A and P301S tau fibrillize with accelerated kinetics compared to WT, with nearly overlapping curves, while P301L aggregates more slowly but remains faster than WT. **C** Quantification of fibrillization half-times shows significantly reduced lag phases for P301L, P301S, and P301A relative to WT (one-way ANOVA with Holm-Sidak multiple comparisons test). **D** Negative-stain transmission electron microscopy confirms mature and abundant fibril morphology by all four proteins, including P301A. Scale bar, 100 nm.

To evaluate the pathogenic potential of the P301A substitution, we expressed and purified four full-length 2N4R tau proteins: WT, P301A, P301L, and P301S. SDS-PAGE confirmed high-purity preparations of all four proteins (Fig. 3A). Kinetic Thioflavin T (ThT) assays revealed that P301A and P301S tau displayed nearly overlapping aggregation kinetics, both markedly accelerated relative to WT, whereas P301L aggregated more slowly but still faster than WT (Fig. 3B). Quantitative analysis of aggregation half-times demonstrated significantly shortened lag phases for P301A, P301S, and P301L compared with WT (Fig. 3C). Transmission electron microscopy confirmed that all four proteins, including P301A, formed fibrillar structures with abundant, mature filament morphology (Fig. 3D). Definitive assessment of variant-specific fibril polymorphs would require higher-resolution structural approaches beyond the scope of this study. Together, these findings establish that P301A tau undergoes rapid, robust fibrillization, comparable to other pathogenic P301 substitutions.

**Fig. 4:**
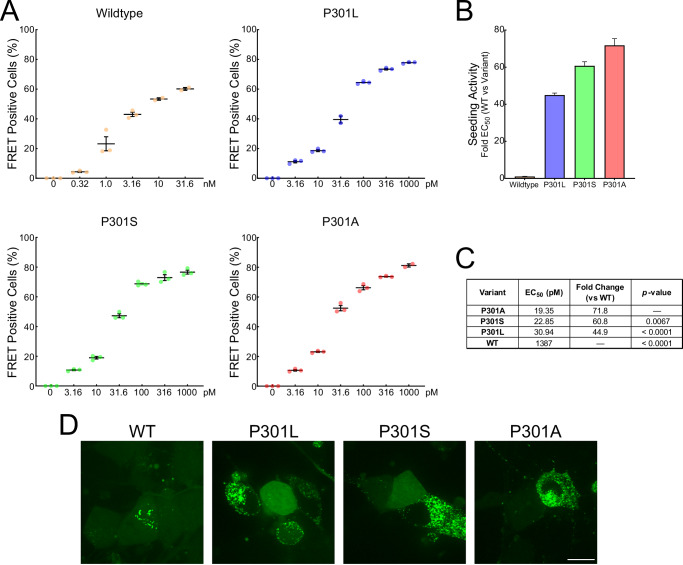
Cell-based seeding shows P301A tau is an exceptionally potent aggregate seed. **A** Dose–response curves from HEK293T tau FRET biosensor cells exposed to fibrils generated from full-length WT, P301L, P301S, or P301A tau. Cells were treated with tau fibrils for 24 h and scored by flow cytometry as % FRET-positive cells. **B** The seeding activity index is calculated for each variant. **C** Potency estimates (EC50) and fold-change vs WT derived from the fits. Statistical analyses were performed with a one-way ANOVA and Holm-Sidak multiple comparisons tests; adjusted *p*-values were calculated from pairwise comparisons using P301A as the reference group to enable comparison among pathogenic variants. **D** Confocal images of iNeuron tau FRET biosensors treated with tau fibril variants (10 nM) for 7 days. The bright intraneuronal foci indicate tau aggregation from exogenously applied tau seeds. Scale bar = 10 µm. In all graphs, the data represent the means ± SEM.

To directly assess the pathogenic seeding capacity of P301A tau, we first performed live-cell imaging of HEK293T tau FRET biosensors (Fig. S2 and Supplementary Movie 1), an assay in which exogenous tau fibrils trigger intracellular aggregation of fluorescently tagged tau reporter proteins^13,14^. Compared with wild-type fibrils, P301A fibrils produced an earlier onset and visibly faster accumulation of tau inclusions, with widespread inclusion formation by ~12 h, whereas WT showed minimal inclusion formation over the same interval. We compared the seeding efficiency of fibrils formed by WT, P301L, P301S, and P301A tau by FRET flow cytometry. Dose–response curves revealed markedly enhanced seeding for all P301 variants relative to WT (Fig. 4A). Expressing the seeding activity as “Fold Change vs WT” (EC₅₀_WT/EC₅₀_variant) showed P301A > P301S > P301L (~72×, ~61×, and ~45× vs WT, respectively), with P301A exhibiting significantly greater seeding potency than P301S and P301L (Fig. 4B). The seeding statistics are summarized using the global 4-parameter logistic model (Fig. 4C). Together, these results demonstrate qualitatively faster and quantitatively stronger cellular seeding by P301A, consistent with a pathogenic gain-of-function similar to other established *MAPT* mutations.

To assess seeding in a more physiologically relevant neuronal context, we next applied tau fibrils (10 nM) to human iNeuron FRET biosensors, a model previously shown to recapitulate intracellular tau aggregation in post-mitotic neurons^15^. All three P301 variants—P301A, P301S, and P301L—robustly induced intraneuronal tau inclusions, whereas WT fibrils produced less aggregation (Fig. 4D). These findings confirm that P301A tau can efficiently seed tau pathology in neurons.

## Discussion

We report, to our knowledge, the first FTD syndrome associated with the *MAPT* P301A substitution. The proband, aged 21, presented with rapidly progressive FTD and marked frontotemporal atrophy with an age and tempo far outside the late-40s to 50 s typical for MAPT-FTLD. This suggests that the P301A variant may cause particularly early or variable-onset FTD. Exome-based and long-read sequencing identified a solitary, rare *MAPT* variant without alternative genetic drivers. In parallel, purified P301A tau showed accelerated fibrillization and robust cellular seeding in biosensor assays, mirroring the patient’s rapid clinical progression. Taken together, these clinical, imaging, and mechanistic data support a pathogenic gain-of-function at this established hotspot and extend the spectrum of disease-causing substitutions beyond P301L/S/T. While inference from a single case requires caution, the combination of early youth, rapid course, and biophysical concordance argues that P301A should be considered pathogenic and should be included in evaluations of early, rapidly progressive FTD.

There was no known family history of young-onset dementia on either side of the pedigree, and neither parent was reported to have cognitive impairment. The proband’s father died of cancer in his 50 s, and several paternal relatives had cancer, but there were no confirmed neurodegenerative cases. The proband’s mother was in her 60 s at the time of presentation and declined genetic testing. Taken together with our sequencing results, these observations raise the possibility that this variant is de novo; however, the absence of parental genotyping and the variant’s rarity preclude definitive assignment.

A large international study demonstrated that age of onset and disease duration in inherited FTD are strongly influenced by the specific *MAPT* mutation. For example, carriers of *MAPT* P301L mutations show phenotypic variability, with a mean age of onset of 53 years and average disease duration of 8 years, whereas *MAPT* P301S mutation carriers present substantially earlier, with an average age of onset of 34 years and a shorter disease course of 5 years^16^. The proband described here presented at an even younger age with a more rapid clinical trajectory than has been reported for most P301L or P301S cases, suggesting that substitution at this codon can give rise to a wide spectrum of disease severity and that P301A may represent an extreme on this continuum.

At the same time, we can not exclude the contribution of environmental modifiers to the unusually early and rapid presentation. The proband’s symptoms emerged following a mild head injury, and traumatic brain injury has been associated with accelerated tau pathology and cognitive decline in experimental models expressing P301S tau^17^. Consistent with this, epidemiologic studies in humans have demonstrated a dose-dependent relationship between prior traumatic or repetitive head injury and earlier symptom onset in behavioral variant FTD^18^. Thus, while our genetic and functional data support a pathogenic role for P301A, head injury may have acted as a disease modifier, accelerating clinical onset or progression.

Given the rapid clinical trajectory, this P301A case meets criteria for a rapidly progressive dementia. Our workup did not identify any vascular, infectious, autoimmune, or neoplastic etiologies. The proband’s initial presentation is less likely to be explained by prion disease, given the absence of myoclonus, visual/cerebellar signs, mutism, or pyramidal/extrapyramidal signs. Further, MRI did not demonstrate diffusion restriction in the cortical and deeper basal ganglia structures, and EEG did not show periodic sharp waves. As her family declined further lumbar punctures, CSF RT-QuIC and 14-3-3 analysis could not be completed.

From a biochemical perspective, tau is a microtubule-associated protein expressed as six adult brain isoforms that differ by inclusion of three or four pseudo-repeats (3R vs 4R) within the repeat domain (RD; residues ~244–365)^19,20^. The RD harbors the amyloid-forming hexapeptide 306–311 (VQIVYK), which nucleates fibril assembly and sits C-terminal immediately to the 301-PGGG-304 turn motif^21,22^. Missense mutations clustering at inter-repeat junctions, especially near the R2-R3 interface encompassing P301 and VQIVYK, shift tau from inert monomers toward seed-competent states and accelerate aggregation in vitro and in cells^23,24^.

Proline uniquely constrains backbone bond angles and stabilizes β-turns. At 301, proline helps form a local β-hairpin that shields the VQIVYK motif from solvent, thereby suppressing spontaneous nucleation. Work by Chen et al. demonstrated that pathogenic substitutions at 301 (e.g., P301L/S) or proline isomerization can destabilize this hairpin, expose VQIVYK, and markedly accelerate aggregation. Conversely, artificially stabilizing the hairpin attenuates aggregation and seeding^23^.

Our findings with P301A fit this framework: replacing proline with alanine removes the rigid pyrrolidine ring, increases local backbone flexibility, and is predicted to weaken the protective hairpin, thereby lowering the barrier to fibrillization and enhancing cellular seeding. Notably, the loss of proline alone may not fully explain pathogenicity—side-chain size, hydrophobicity, and polarity likely tune the conformational ensemble—consistent with our observation that P301A can exceed P301S and P301L in seeding assays at equivalent doses. Looking ahead, a deep mutational scan at and around P301 could systematically map how side-chain chemistry and local structure cooperate to regulate exposure of VQIVYK, and thereby aggregation and toxicity.

## Methods

Clinical evaluation was performed at the University of California, San Francisco, Memory and Aging Center. The participant was enrolled in a research project on FTD at UCSF for which she provided written informed consent in accordance with the Declaration of Helsinki. The San Francisco General Hospital Institutional Review Board approved the study; the reference number is 10-03946. Exome sequencing was conducted at the UCSF Genomic Medicine Laboratory. MRI scans were obtained on a 3-T scanner.

### Exome sequencing

Exonic regions were targeted in extracted genomic DNA from submitted specimens using the Twist Bioscience Human Comprehensive Exome and Mitochondrial Panel. Targeted regions were sequenced using the Illumina sequencing system with 100 bp paired-end reads. The resulting DNA sequences were mapped to and analyzed in comparison with the published human genome (UCSC hg38 reference sequence). Single-nucleotide variants were filtered, ranked, and annotated using Moon (Invitae Corporation). For the mitochondrial genome, the revised Cambridge Reference Sequence was used. All reported variants were confirmed using a combination of long-range PCR and sequencing. The mean depth of coverage was 149×, and the quality threshold was 99.57% at ≥25×.

### Long-read sequencing

Long-read genome sequencing was performed as previously described^25^. Briefly, high–molecular-weight genomic DNA from the proband was sequenced using PacBio HiFi (CCS) chemistry on a Revio instrument (Pacific Biosciences). SMRTbell libraries were prepared using the SMRTbell Template Prep Kit 3.0, size-selected with AMPure beads, and sequenced on two Revio SMRT Cells (2-h pre-extension, 30-h movie time). CCS reads were generated with CCS v8.2.0, adapter-trimmed (pbtrim v1.1.0), and demultiplexed (lima v2.11.0), yielding ~30× HiFi coverage. HiFi reads were aligned to the GRCh38 no-alt reference using pbmm2 v1.17.0. SNVs and indels were called with DeepVariant v1.8.0 and phased using WhatsHap v2.4. Structural variants were identified with pbsv v2.10.0. Variant calls were annotated using an in-house analysis pipeline incorporating gnomAD population frequencies and standard clinical variant-interpretation resources. Structural variants were additionally filtered using bcftools and prioritized using SvAnna v1.0.4. Repeat expansions were assessed in a curated set of disease-associated tandem-repeat loci using pbsv calls, IGV visualization, and TRGT/TRVZ (hg38 reference, BED-based repeat catalog).

### Protein expression, purification, and fibrillization

Recombinant human full-length 2N4R tau (WT, P301A, P301L, P301S, and corresponding cysteine-free “Cys0” variants with C291A/C322A substitutions) was cloned into pET-23a vectors and expressed in *E. coli* BL21-CodonPlus cells (Agilent; 230255) as an N-terminal Hisx6-SUMO fusion protein. *E. coli* were transformed with the expression plasmids, and 5-mL LB starter cultures containing 100 µg/ml carbenicillin were grown overnight. These were used to inoculate 1.5 L LB/carbenicillin, which was cultured at 37 °C for ~3 h until OD_600_ = 0.6–0.7. Cultures were then cooled to 16 °C for 1 h before induction with 1 mM IPTG, followed by overnight expression at 16 °C for 16 h. Cells were harvested by centrifugation at 10,000 × *g* for 10 min at 4 °C. The cell pellet was resuspended in lysis buffer (100 mM Tris, pH 8.0, 150 mM NaCl) and lysed by sonication. The lysate was clarified by centrifugation (20,000 × *g*, 30 min, 4 °C), and the supernatant was applied to a 2 mL cobalt Talon affinity resin column (Takara; 635655). After washing with 40 mL lysis buffer, the tau fusion protein was eluted with 10 mL of elution buffer (100 mM Tris, pH 8.0, 150 mM NaCl, 500 mM imidazole). The eluate was supplemented with 1 mM DTT and 5 mM EDTA. To remove the SUMO tag, the fusion protein was placed in a 10-kDa MWCO dialysis bag with ULP1 protease (1 mg per 25 mg fusion protein) and dialyzed overnight (4 °C) against 100 mM Tris, pH 8.0, 250 mM NaCl, 5 mM DTT. The cleaved protein was concentrated to ~3 mL using a Vivaspin Turbo 10-kDa ultrafilter (Sartorius; VS15TR023) and purified by size-exclusion chromatography on a Superdex 75 pg 16/600 column (Cytiva; 28989333) equilibrated in 50 mM sodium phosphate buffer, pH 7.5, 100 mM NaCl. Fractions containing pure monomeric tau were combined, concentrated to 2 mg/ml, flash frozen in liquid nitrogen, and stored at −80 °C. The Cys0 constructs were used in Thioflavin T assays to prevent disulfide-mediated dimerization and off-target fibrillization as previously described^14,26,27^.

### Thioflavin T assay

Kinetic Thioflavin T (ThT) assays were performed as previously described^26^ with minor modifications. Reactions contained 9 μM Cys0 tau monomer, 50 μM Thioflavin T (Sigma-Aldrich; T3516-5G), and 0.5 mM TCEP (pH 6.7; GoldBio) in 1× PBS in black 384-well plates (Greiner, 781209). Following a 30-min incubation at 37 °C, aggregation was initiated with 8 µM heparin (Sigma, H3393). Plates were continuously shaken at 37 °C while fluorescence was monitored (Ex 440 nm; Em 485 nm) for 72 h on a SpectraMax iD5 plate reader. The kinetic curves were analyzed in GraphPad Prism 10. For each experiment, conditions were analyzed in technical triplicate. All experiments were performed three separate times (biological replicates), and a representative experiment is depicted in the figures.

### Negative-stain transmission electron microscopy

Fibrillization reactions were assessed by negative staining and transmission electron microscopy. Fibrils were diluted to 50 µg/mL in 10 mM HEPES, 100 mM NaCl, and 1 mM DTT at pH 7.4. To a glow-discharged formvar/carbon-coated 400-mesh copper grid (Ted Pella; 01754-F), 5 µL of sample was applied for 1 min, washed with deionized water, and then stained with 30 µL of 2% (w/v) uranyl formate for 1 min. Samples were imaged using a FEI Tecnai G2 Spirit Biotwin operating at 120 kV with a Gatan Rio camera.

### Tau seeding assay and FRET flow cytometry

Tau seeding activity was measured using HEK293T tau FRET biosensor cells^13,14^. For live-cell imaging, fibrils were transduced with Lipofectamine 3000 (ThermoFisher) and imaged on an Opera Phenix for 48 h using a 488 nm laser and a 500–550 nm emission filter or Crest X-Light-V2 spinning disk confocal (Crest Optics). Image analysis was performed using Revity’s Harmony software. First, cell boundaries were defined by using the built-in cell segmentation function on the FRET acceptor channel (405 nm/500–550 nm). Next, puncta were identified also using the FRET acceptor channel (405 nm/500–550 nm) using spot search restricted to the intracellular region within the segmented cell perimeter. Finally, the cells and puncta were counted in each image, and the average number of cells and puncta in each well (four images) was exported. The average number of puncta per cell in each well was calculated for each treatment condition and plotted against the time since the fibrils were administered. The images from a representative field were also combined to generate a seeding movie for each treatment condition.

For FRET flow cytometry, cells were harvested with 0.05% Trypsin (Gibco) and analyzed on a CytoFLEX cytometer (Beckman Coulter) using CytExpert software v2.3.1.22. The percentage of FRET-positive cells was analyzed using FlowJo v.10.9, and nonlinear regression analyses (log[agonist] vs. normalized response, variable slope) were performed in GraphPad Prism 10.0. For each experiment, 10,000 cells per replicate were collected, and each condition was analyzed in technical triplicate. All experiments were performed three separate times (biological replicates), and a representative experiment is depicted in the figures.

## Supplementary information

Supplementary Information

Supplementary Movie 1

## Data Availability

The datasets generated and/or analyzed during the current study are not publicly available due to the complexity and size of the raw experimental data and the inclusion of data derived from patient-related biological materials, which require controlled access, but are available from the corresponding author on reasonable request.

## Code availability

There was no code for this project.
