# Supplementary material for: Early onset rapidly progressive frontotemporal dementia due to a novel MAPT P301A variant with functional validation of pathogenicity
Source: NPJ Dement. 2026 May 25;2(1):38. doi: 10.1038/s44400-026-00068-w (PMC13201157; doi:10.1038/s44400-026-00068-w)
Supplement: Supplementary file 1 — Supplementary Information [file 44400_2026_68_MOESM1_ESM.pdf]

Figure S1

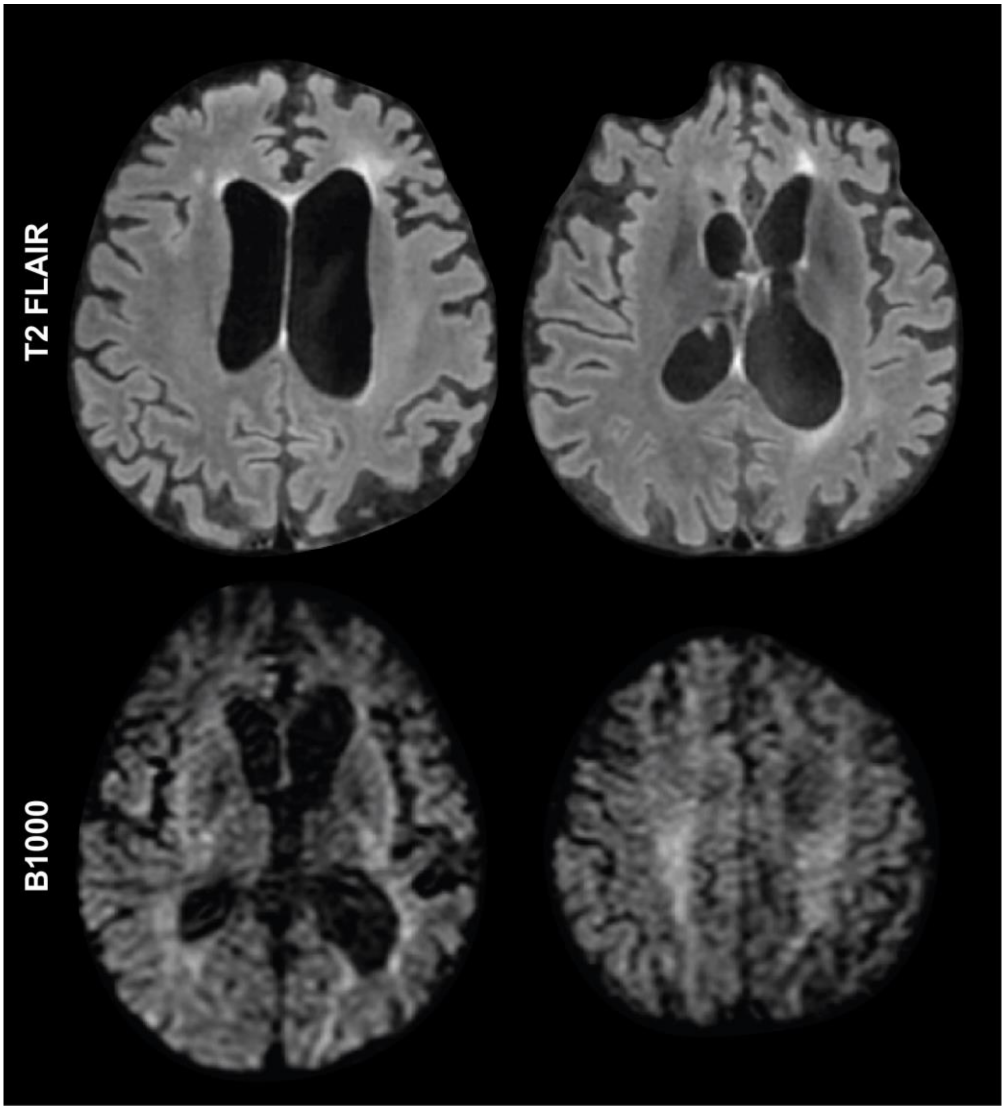

Figure S1. Brain MRI: Follow-up axial T2 FLAIR MRI (top) demonstrated hyperintensities in the periventricular and bilateral subcortical white matter. B1000 diffusion-weighted images (bottom) show no evidence of cortical ribboning or diffusion restriction.

Figure S2

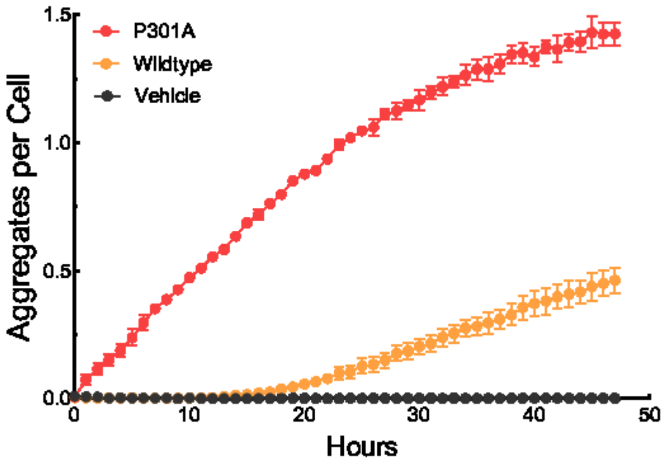

Figure S2. Kinetic Analysis of Cellular Seeding: Live-cell imaging analysis of HEK293T tau FRET biosensors treated with 10 nM of tau P301A fibrils, WT fibrils, or vehicle. Cells were imaged every hour for 48 hr. The data represent the means ± SEM.

Supplementary Movie 1. Live-Cell Imaging of Cellular Seeding: Live-cell imaging of HEK293T tau FRET biosensors treated with 10 nM of tau WT fibrils (left) or P301A fibrils (right). Cells were imaged every hour for 48 hr. The bright intracellular foci represent induced tau aggregation from exogenously applied tau seeds. Scale bar = 20 μm.
